# m^6^A-induced lncRNA MALAT1 aggravates renal fibrogenesis in obstructive nephropathy through the miR-145/FAK pathway

**DOI:** 10.18632/aging.102950

**Published:** 2020-03-23

**Authors:** Peihua Liu, Bo Zhang, Zhi Chen, Yao He, Yongchao Du, Yuhang Liu, Xiang Chen

**Affiliations:** 1 Department of Urology, Xiangya Hospital, Central South University, Changsha 410008, Hunan, PR China

**Keywords:** renal fibrosis, TGF-β1, MALAT1, dihydroartemisinin, mA ^6^

## Abstract

Renal fibrosis is a key factor in chronic kidney disease (CKD). Long non-coding RNAs (lncRNAs) play important roles in the physiological and pathological progression of human diseases. However, the roles and underlying mechanisms of lncRNAs in renal fibrosis still need to be discovered. In this study, we first displayed the increased lncRNA metastasis-associated lung adenocarcinoma transcript 1 (MALAT1) expression in renal fibrosis in patients with obstructive nephropathy (ON). Then we found that transforming growth factor beta 1 (TGF-β1) induced epithelial-mesenchymal transition (EMT) and extracellular matrix (ECM) protein deposition, which promoted the viability, proliferation and migration of human renal proximal tubular epithelial (HK2) cells. Next, MALAT1/miR-145/focal adhesion kinase (FAK) pathway was confirmed to play an importment role in TGF-β1-induced renal fibrosis. In addition, the MALAT1/miR-145/FAK pathway was involved in the effect of dihydroartemisinin (DHA) on TGF-β1-induced renal fibrosis *in vitro* and *in vivo*. Furthermore, m^6^A methyltransferase methyltransferase-like 3 (METTL3) was shown to be the main methyltransferase of m^6^A modification on MALAT1.

## INTRODUCTION

As a global epidemic, chronic kidney disease (CKD) has severe personal and societal consequences [1]. Obstructive nephropathy (ON) is the renal disease caused by impaired flow of urine or tubular fluid, which refers to the presence of structural or functional changes in the urinary tract that impede the normal flow of urine [2]. Obstructive uropathy, which can lead to ON without timely intervention, is the main cause of CKD. Common obstructive factors include stones, blood clots, tumours and lymphadenopathy [3]. However, a considerable number of patients with ON have clinical characteristics that are difficult to detect; these patients are often symptomless in the early stages and have delitescent pathogenetic conditions.

Fibrosis is a hallmark of CKD and affects both the glomeruli and the tubules as well as causes renal vasculature alterations [4]. Considering the role of renal fibrosis in CKD with ON, we believe that understanding the formation, reversal and underlying mechanism of renal fibrosis could provide valuable insights and opportunities for improving monitoring techniques and therapeutic interventions for CKD caused by ON or even for other diseases.

Renal fibrosis is characterized by epithelial-mesenchymal transition (EMT), excess extracellular matrix (ECM) deposition and fibroblast and inflammatory cell accumulation in the interstitium [5–7]. Through EMT, tubular epithelial cells become myofibroblasts, which possess enhanced capacities for cell proliferation, motility, contraction and excess ECM deposition, thus leading to fibrosis [8, 9]. Increasing evidence suggests that numerous genes, including cytokines, growth factors, metabolic toxins, and stress molecules, are involved in the progression of renal fibrosis [10].

Transforming growth factor-β1 (TGF-β1), a well-studied profibrogenic cytokine, is synthesized by various cell types, such as epithelial cells, lymphocytes, platelets, fibroblasts, astrocytes, macrophages and kidney cells [11]. Previous studies have documented that TGF-β1 plays an important role in the progression of CKD because it promotes EMT and ECM deposition [12, 13]. Furthermore, as a multifunctional growth factor, TGF-β1 plays important roles in a variety of cellular processes, including growth, proliferation, cell cycle progression, development, differentiation, migration, invasion and inflammation [14, 15]. TGF-β1 exerts its function by modulating the TGF-β/Smad pathway, and it could be the master regulator of fibrosis [14, 16].

In recent years, a growing number of long non-coding RNAs (lncRNAs) have attracted increasing attention from researchers. LncRNAs are evolutionarily conserved non-protein-coding transcripts longer than 200 nucleotides [17, 18]. Emerging evidence has shown that lncRNAs play important roles in the physiological and pathological progression of human diseases [19–26]. Several lncRNAs, such as H19, MEG3, TUG1 and PVT1, are reportedly involved in CKD [27–30]. Thus, better understanding the roles and mechanisms of lncRNAs in the physiological and pathological progression of CKD will help elucidate possible opportunities for therapeutic CKD interventions. Metastasis associated lung adenocarcinoma transcript 1 (MALAT1) is involved in frequent tumours including renal cell carcinoma, bladder cancer, prostate cancer, breast cancer and so on [31]. Furthermore, MALAT1 can be involved in several other pathophysiological conditions such as myogenesis and synaptogenesis [32].

Focal adhesion kinase (FAK) is a protein tyrosine kinase that contributes to cancer progression and can play roles in EMT, radio-resistance, DNA damage repair and tumor immune evasion as well [33].

N6-methyladenosine (m^6^A) modification is the most abundant internal modification in eukaryotes [34]. The successive discoveries of m^6^A-binding proteins (“readers”), adenosine methyltransferases (“writers”) and m^6^A demethylating enzymes (“erasers”) demonstrated that the m^6^A modification is a reversible process [35–37]. While m^6^A modifications are known to modulate RNAs, including mRNAs and lncRNAs [34], the mechanisms of m^6^A modification in lncRNAs still need to be investigated. As the core methyltransferase subunit, methyltransferase-like 3 (METTL3) is the catalytic subunit which selectively methylates the GAC and AAC sequences in synthetic single-stranded RNA in vitro [38].

Recently, although major advances in understanding the physiological and pathological processes of renal fibrosis have occurred, efforts to curb the progression of CKD have also been made [39]. Many compounds exert either a direct or indirect antifibrotic effect [39]. For example, pirfenidone (PFD) has been demonstrated to play an important role in delaying the progression of fibrosis [39–41]. Dihydroartemisinin (DHA), an effective antimalarial drug derived from the natural small-molecule compound artemisinin, has attracted substantial attention in recent years for its pharmacological activities, such as its antibacterial and antifibrotic properties [42]. However, few reports about the role of lncRNAs and the antifibrotic effects of these drugs have been published.

In this study, we investigated the mechanism of MALAT1 in ON-induced renal fibrosis. Even if this is not the first study for MALAT1 in renal fibrosis, our study indeed further revealed the role of MALAT1 in ON-induced renal fibrosis and the mechanism by which METTL3 positively regulates MALAT1. Furthermore, we explored whether DHA, an effective antimalarial drug, attenuates renal fibrosis through MALAT1 [42].

## RESULTS

### MALAT1 expression was upregulated in renal fibrotic tissues in patients with ON

**Figure 1:**
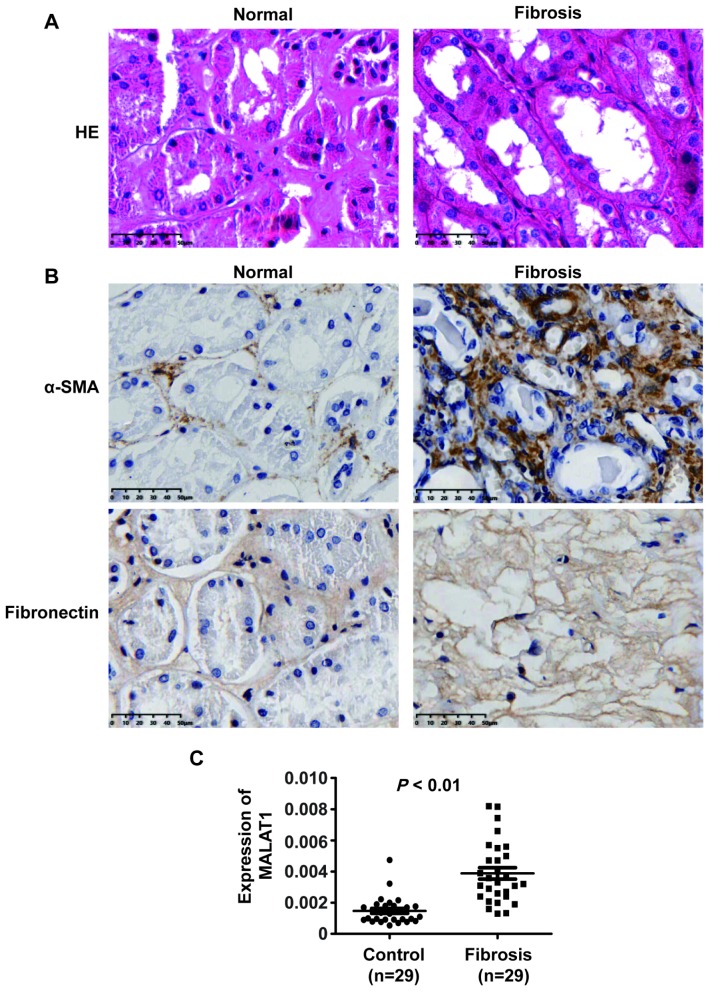
**MALAT1 expression was upregulated in renal fibrotic tissues in patients with ON.** (**A**) HE analyses of kidney tissue with severe hydronephrosis from obstructive stones and normal tissue. (**B**) IHC analyses of α-SMA and ECM deposition in samples from patients with renal fibrosis. (**C**) qPCR analysis of MALAT1 expression in samples from patients with renal fibrosis].

MALAT1 has been shown to play significant roles in many human diseases. To dissect whether MALAT1 is involved in the progression of renal fibrosis in patients with ON, we measured MALAT1 expression in renal fibrotic tissues in patients with ON. Haematoxylin and eosin (HE) staining analyses showed kidney tissue with severe hydronephrosis from obstructive stones and normal tissue (Figure 1A).

Furthermore, immunohistochemistry (IHC) staining analyses revealed that α-SMA and ECM deposition were increased in samples from patients with fibrosis compared with those in normal tissue samples (Figure 1B). qPCR analyses illustrated that MALAT1 expression was increased in renal fibrosis tissues compared to that in normal tissues (Figure 1C). Collectively, these observations indicated that MALAT1 was involved in fibrosis.

### TGF-β1 induced fibrosis via upregulating MALAT1 expression in HK2 cells

Of mention above, we displayed an increased expression of MALAT1 in clinicopathological specimen of renal fibrosis. We wonder whether MALAT1 exerted an important role in fibrosis and further, what is the underlying molecular mechanism of the role.

**Figure 2:**
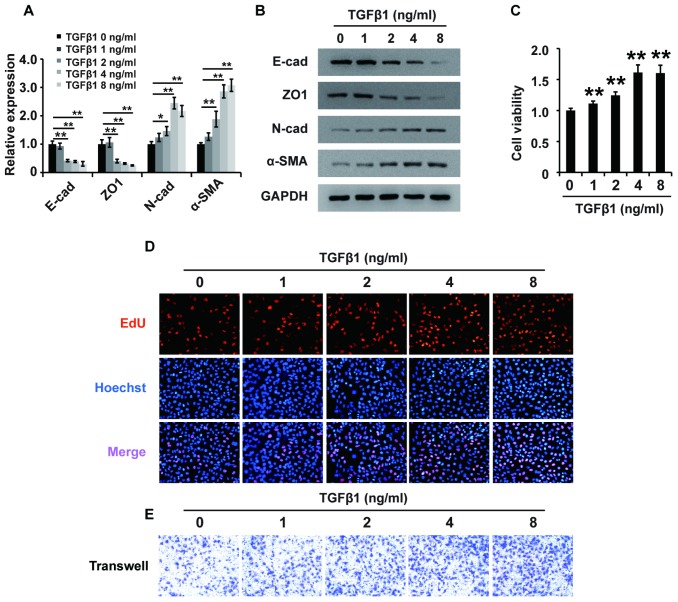
**TGF-β1 induces fibrosis in HK2 cells.** (**A**) qPCR analyses of E-cadherin (E-cad), ZO1, N-cadherin (N-cad) and α-SMA expression in HK2 cells treated with TGF-β1 at different concentrations for approximately 48 h. (**B**) Western blot analyses of E-cad, ZO1, N-cadherin and α-SMA expression in HK2 cells treated with TGF-β1 at different concentrations for approximately 48 h. GAPDH was used as a control. (**C**–**E**) CCK-8, EdU and cell migration analyses (transwell) of the viability, proliferation and migration of HK2 cells treated with TGF-β1 at different concentrations for approximately 48 h. *P < 0.05 and **P *<* 0.01.

To determine the role of TGF-β1 in fibrosis, we treated HK2 cells with different concentrations of TGF-β1 for 48 h and then performed qPCR, western blot, CCK-8, EdU and cell migration assays. qPCR and western analyses showed that TGF-β1 downregulated the expression of E-cadherin and ZO1 and upregulated the expression of N-cadherin and α-SMA in HK2 cells (Figure 2A and 2B). These results demonstrated that TGF-β1 induced EMT in HK2 cells. Then, CCK-8 assays showed that TGF-β1 enhanced the viability of HK2 cells (Figure 2C). Furthermore, EdU assays demonstrated that TGF-β1 promoted the proliferation of HK2 cells (Figure 2D). Cell migration assays illustrated that TGF-β1 strengthened the cell migration potential of HK2 cells (Figure 2E). In summary, these data revealed that TGF-β1 promoted EMT and enhanced the viability, proliferation and migration potential of HK2 cells.

**Figure 3:**
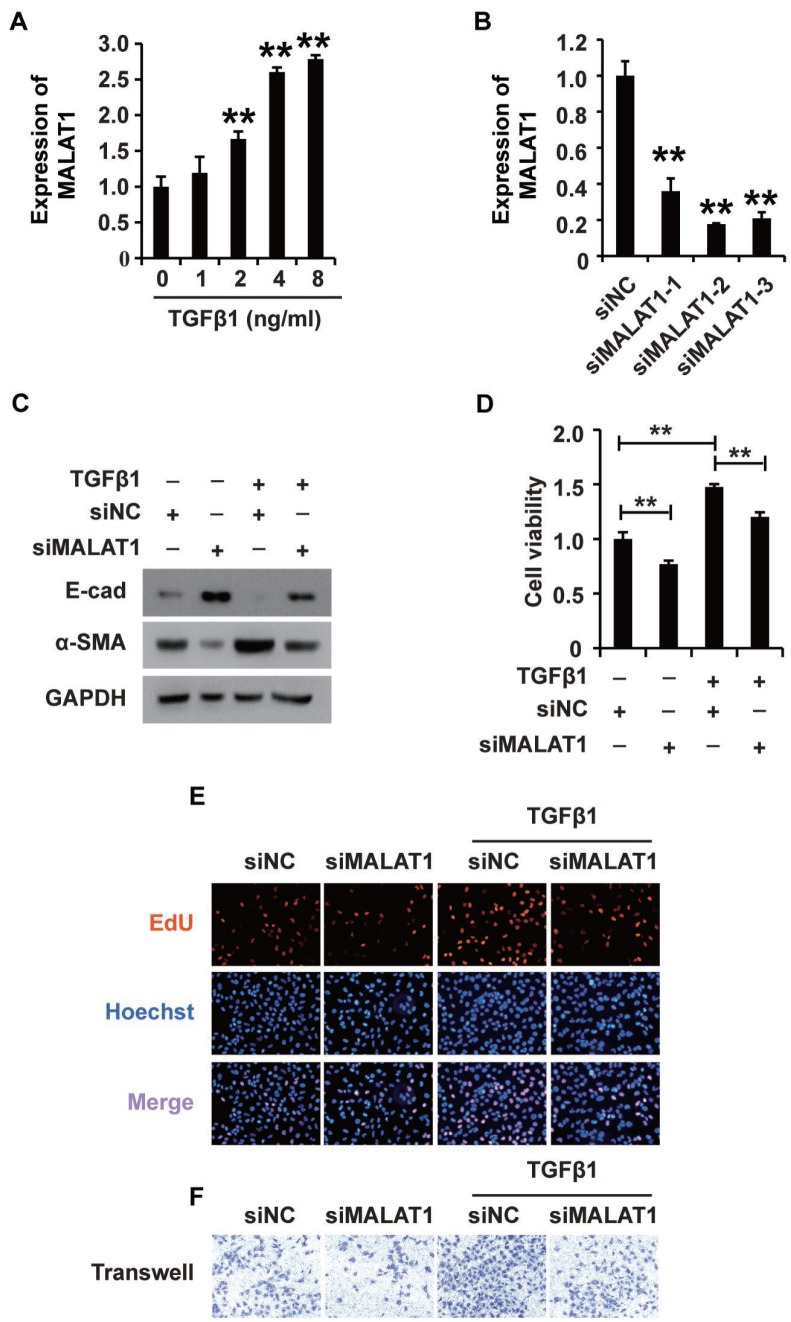
**TGF-β1 induces fibrosis via upregulating MALAT1 expression in HK2 cells.** (**A**) qRT-PCR analysis of MALAT1 expression in HK2 cells treated with TGF-β1. (**B**) qRT-PCR analysis of MALAT1 expression in HK2 cells transfected with siMALAT1 or siNC for approximately 48 h. (**C**) Western blot analyses of E-cad, α-SMA and GAPDH expression in HK2 cells receiving different treatments. (**D**–**F**) CCK8, EdU and cell migration analyses of the viability, proliferation and migration of HK2 cells receiving different treatments. After pretransfection with siMALAT1 or siNC for 24 h, HK2 cells were treated with 4 ng/mL TGF-β1 for another 48 h. GAPDH was used as a control. *P < 0.05 and **P < 0.01.

Research has demonstrated that MALAT1 plays extensive roles in a variety of cellular processes [36]. We proposed that MALAT1 might play an important role in mediating the effects of TGF-β1 in HK2 cells. To elucidate the possible role of MALAT1, we first employed qPCR to detect its expression in HK2 cells treated with TGF-β1, revealing that TGF-β1 increased MALAT1 expression in HK2 cells (Figure 3A). Then, we used three siRNAs specific to MALAT1 to knockdown its expression, and qPCR analyses illustrated that all three siRNAs could effectively inhibit MALAT1 expression (Figure 3B). siMALAT1-2 was subsequently chosen for further functional research. Excitingly, western blot analysis showed that inhibiting MALAT1 reversed TGF-β1-induced EMT (Figure 3C). Furthermore, CCK-8, EdU and cell migration analyses demonstrated that knocking down MALAT1 inhibited the viability, proliferation and migration potential of HK2 cells treated with TGF-β1 (Figure 3D–3F). In addition, overexpression of MALAT1 can induce the EMT, enhance the cell viability, promote the cell proliferation and migration potential of HK2 cells (Supplementary Figure 1).

Together, these results suggest that TGF-β1 plays a role in fibrosis by activating MALAT1 expression in HK2 cells.

### MALAT1 functions by acting as a miR-145 sponge in HK2 cells treated with TGF-β1

**Figure 4:**
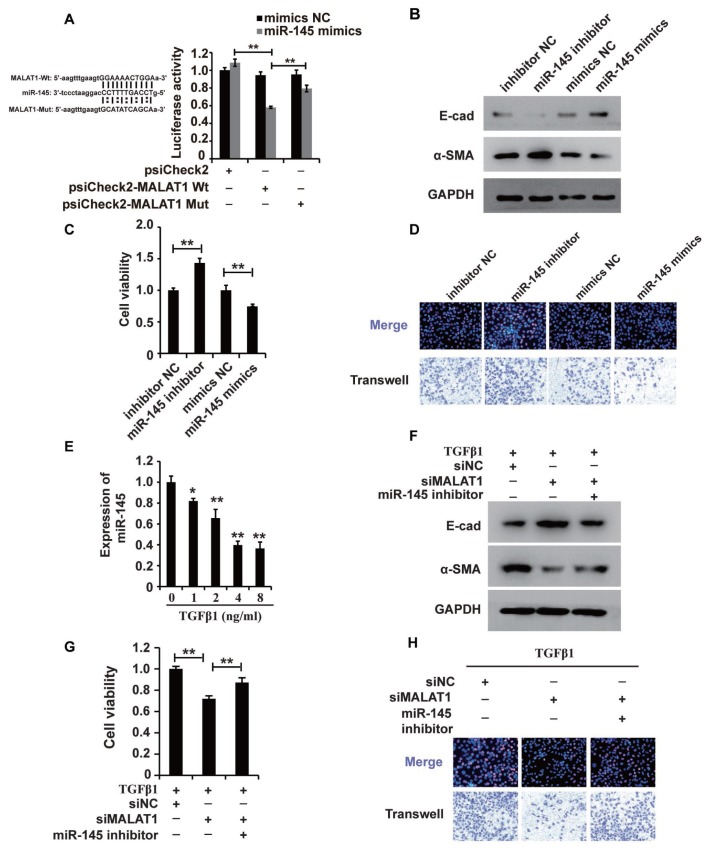
**MALAT1 acts as a miR-145 sponge in HK2 cells treated with TGF-β1.** (**A**) Luciferase reporter analysis of the binding between miR-145 and predicted MALAT1 binding sites. (**B**) Western blot analyses of E-cad, α-SMA and GAPDH expression in HK2 cells transfected with miR-145 mimics, miR-145 inhibitors and their control RNAs. (**C** and **D**) CCK8, EdU and cell migration analyses of the viability, proliferation and migration of HK2 cells transfected with miR-145 mimics, miR-145 inhibitors and their control RNAs. (**E**) qPCR analysis of miR-145 expression in HK2 cells treated with different concentrations of TGF-β1 for 48 h. (**F**) Western blot analyses of E-cadherin, α-SMA and GAPDH expression in HK2 cells receiving different treatments. (**G** and **H**) CCK8, EdU and cell migration analyses of the viability, proliferation and migration of HK2 cells receiving different treatments. GAPDH and U6 were used as controls. *P < 0.05 and **P < 0.01.

Recently, studies have demonstrated the broad applicability of the ceRNA hypothesis to the lncRNA mechanism of action [44]. To examine the mechanism of MALAT1, we systematically analysed its potential miRNA binding sites using online software, which revealed potential miR-145 binding sites. To confirm the binding abilities of the sites identified, we used dual-luciferase reporter. The luciferase activity was decreased in cells cotransfected with wild-type MALAT1 and miR-145 mimics but was restored in cells cotransfected with mutant MALAT1 and miR-145 mimics (Figure 4A), demonstrating that MALAT1 could bind miR-145. Furthermore, the results of RIP showed that MALAT1 and miR-145 were more abundant in the Ago2 pellet than in the control IgG pellet (Supplementary Figure 2).

Then, we used western blotting to examine the role of miR-145 in HK2 cells. In HK2 cells, miR-145 mimics inhibited EMT, and a miR-145 inhibitor promoted EMT (Figure 4B). Furthermore, CCK-8, EdU and cell migration analyses illustrated that the miR-145 mimics inhibited the cell viability, proliferation and migration, while the miR-145 inhibitor promoted the migration of HK2 cells (Figure 4C and 4D).

Given that MALAT1 could bind miR-145 and that miR-145 plays important roles in HK2 cells, we proposed that miR-145 was associated with the functions of MALAT1 in HK2 cells treated with TGF-β1. qPCR and western blot analyses showed that repressing miR-145 restored the siMALAT1-induced inhibition of EMT in HK2 cells treated with TGF-β1 (Figure 4E and 4F). Furthermore, CCK-8, EdU and cell migration analyses illustrated that the miR-145 inhibitor restored the siMALAT1-induced inhibition of cell viability, proliferation and migration, while the miR-145 inhibitor promoted those processes in HK2 cells (Figure 4G and 4H). In summary, these observations revealed that MALAT1 functions by acting as a miR-145 sponge in HK2 cells treated with TGF-β1.

### miR-145 plays a role in regulating FAK expression in HK2 cells treated with TGF-β1

**Figure 5:**
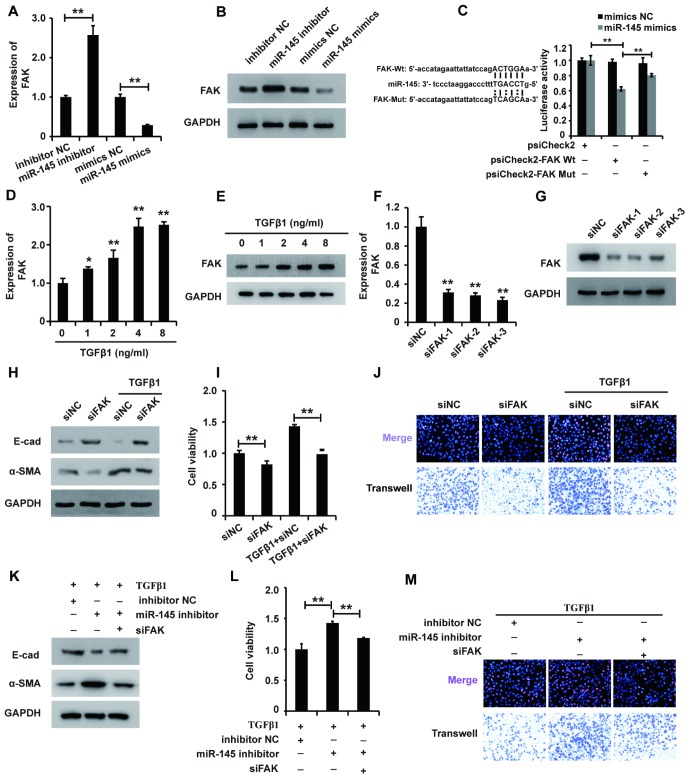
**miR-145 plays a role in regulating FAK expression in HK2 cells treated with TGF-β1.** (**A** and **B**) qRT-PCR and western blot analyses of FAK expression in HK2 cells transfected with miR-145 mimics, miR-145 inhibitors and their control RNAs. (**C**) Luciferase reporter analysis of the binding between miR-145 and predicted binding sites in FAK. (**D** and **E**) qRT-PCR and western blot analyses of FAK expression in HK2 cells treated with TGF-β1 (**F** and **G**). qRT-PCR and western blot analyses of FAK expression in HK2 cells transfected with siFAK or siNC for approximately 48 h. (**H**) Western blot analyses of E-cad, α-SMA and GAPDH expression in HK2 cells receiving different treatments. (**I** and **J**) CCK8, EdU and cell migration analyses of the viability, proliferation and migration of HK2 cells receiving different treatments. (**K**) Western blot analyses of E-cad, α-SMA and GAPDH expression in HK2 cells receiving different treatments. (**L** and **M**) CCK8, EdU and cell migration analyses of the viability, proliferation and migration of HK2 cells receiving different treatments. GAPDH was used as a control. *P < 0.05 and **P < 0.01.

Given the mode of miRNA action, we systematically sought the potential targets of miR-145 using online software and identified FAK as a potential target. Thus, we wondered whether miR-145 exerted its function via inhibiting FAK expression in HK2 cells treated with TGF-β1. qRT-PCR and western blot analyses showed that miR-145 mimics downregulated FAK expression, while the miR-145 inhibitor upregulated FAK expression (Figure 5A and 5B). To further confirm that FAK was a functional target of miR-145, we employed the dual-luciferase reporter assay to investigate the binding between FAK and miR-145. The luciferase activity was decreased in cells co-transfected with wild-type FAK and miR-145 mimics, but restored in cells co-transfected with mutant FAK and miR-145 mimics (Figure 5C). Taken together, these observations suggest that activating miR-145 downregulates FAK in HK2 cells.

Then, we sought to dissect the role of FAK in HK2 cells. qPCR and western blot analyses demonstrated that TGF-β1 promoted FAK expression in HK2 cells (Figure 5D and 5E), suggesting that FAK was associated with the function of TGF-β1 in HK2 cells. To elucidate the possible role of FAK in HK2 cells, we first designed siRNAs specific to FAK and transfected them into HK2 cells. qPCR and western blot analyses showed that all three FAK siRNAs could effectively inhibit FAK expression, and siFAK-1 was chosen for further research (Figure 5F and 5G). Western blot analysis showed that inhibition of FAK reversed the TGF-β1-induced EMT (Figure 5H). Furthermore, CCK-8, EdU and cell migration analyses demonstrated that FAK knockdown inhibited the viability, proliferation and migration potential of HK2 cells treated with TGF-β1 (Figure 5I and 5J). In summary, these results suggested that FAK could be associated with the function of TGF-β1 in HK2 cells.

Given that activating miR-145 downregulated FAK expression and that FAK affected the function of TGF-β1 in HK2 cells, we proposed that miR-145 exerted its function via inhibiting FAK expression in HK2 cells treated with TGF-β1. Western blot analysis showed that repression of FAK restored miR-145-induced EMT in HK2 cells treated with TGF-β1 (Figure 5K). Furthermore, the CCK-8, EdU and cell migration analyses illustrated that repressing FAK restored the miR-145-induced promotion of the viability, proliferation and migration of HK2 cells treated with TGF-β1 (Figure 5L and 5M). In summary, these results show that miR-145 plays a role in regulating FAK expression in HK2 cells treated with TGF-β1.

### MALAT1 regulates the MALAT1/miR-145/FAK axis in HK2 cells treated with TGF-β1

**Figure 6:**
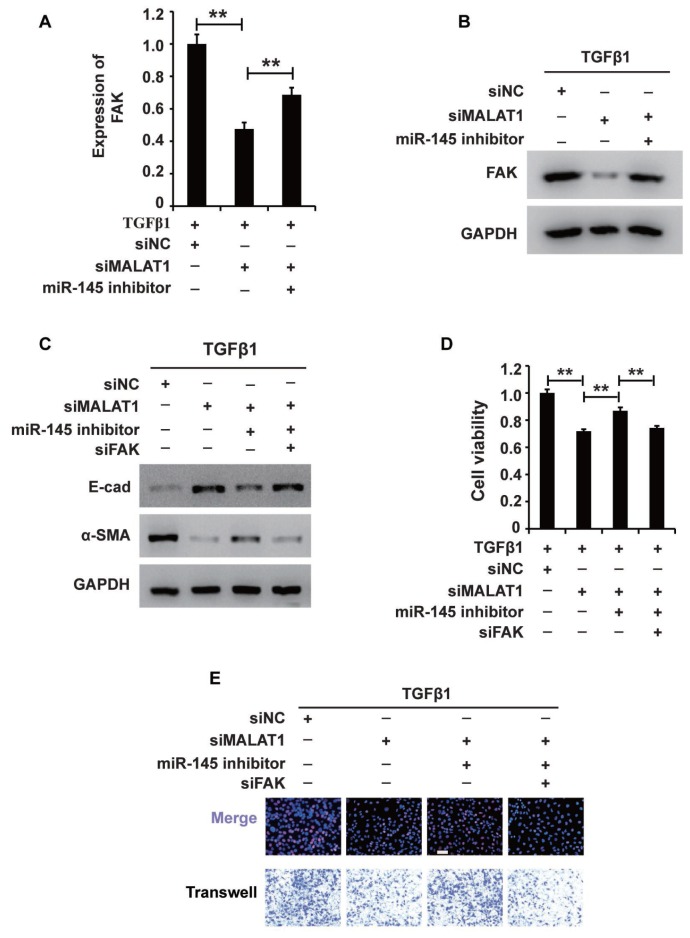
**MALAT1 regulates the MALAT1/miR-145/FAK axis in HK2 cells treated with TGF-β1.** (**A** and **B**) qRT-PCR and western blot analyses of FAK expression in HK2 cells treated with TGF-β1 alone or together with siMALAT1, a miR-145 inhibitor or a NC. (**C**) Western blot analyses of E-cadherin, vimentin, α-SMA and collagen I expression in HK2 cells receiving different treatments. (**D** and **E**) CCK8, EdU and cell migration analyses of the viability, proliferation and migration of HK2 cells receiving different treatments. GAPDH was used as a control. *P < 0.05 and **P < 0.01.

Notably, we found that activating MALAT1 downregulated miR-145 expression and that activating miR-145 downregulated FAK expression in HK2 cells treated with TGF-β1. Thus, we proposed that MALAT1 might function via the MALAT1/miR-145/FAK axis. To test this hypothesis, we first measured whether activating MALAT1 could increase FAK expression via sponging miR-145. qRT-PCR and western blot analyses demonstrated that inhibiting MALAT1 downregulated FAK expression, and this effect was restored by a miR-145 inhibitor (Figure 6A and 6B). These results demonstrated that activating MALAT1 could increase FAK expression by sponging miR-145 in HK2 cells treated with TGF-β1.

Then, we aimed to examine the role of the MALAT1/miR-145/FAK axis in HK2 cells treated with TGF-β1. Western blot analysis revealed that increasing MALAT1 and FAK and decreasing miR-145 activated EMT in HK2 cells treated with TGF-β1 (Figure 6C). In addition, CCK-8, EdU and cell migration analyses illustrated that upregulation of the MALAT1 and FAK and downregulation of miR-145 activated the viability, proliferation and migration potential of HK2 cells treated with TGF-β1 (Figure 6D and 6E). In summary, these results showed that MALAT1 sponges miR-145 and thereby increases FAK expression in HK2 cells treated with TGF-β1.

### The MALAT1/miR-145/FAK axis is involved in the antifibrotic effect of DHA on TGF-β1-induced renal fibrosis

**Figure 7:**
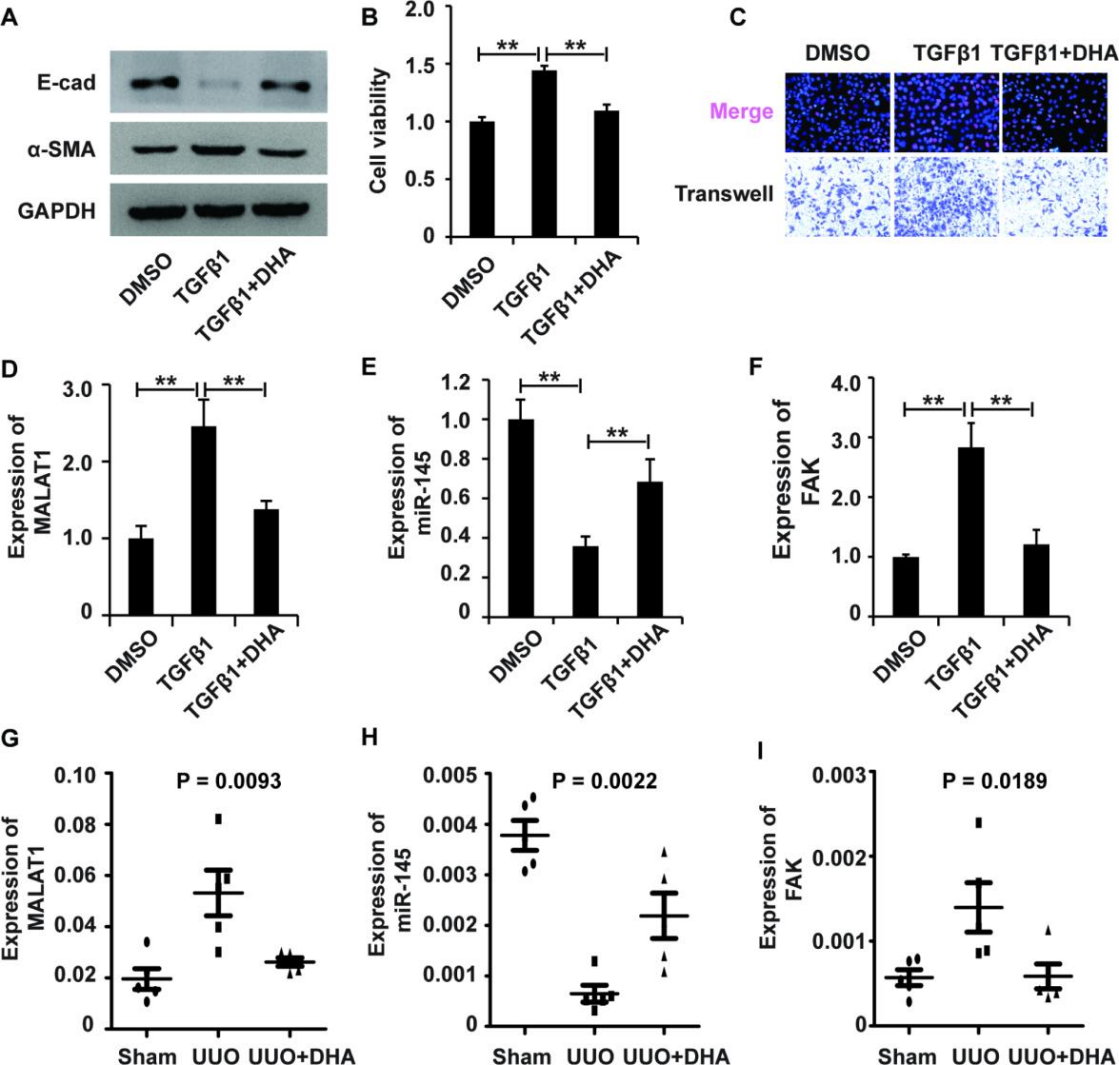
**The MALAT1/miR-145/FAK axis is involved in the mechanism of fibrosis treatment drugs.** (**A**) Western blot analyses of E-cad, α-SMA and GAPDH expression in HK2 cells receiving different treatments. (**B** and **C**) CCK8, EdU and cell migration analyses of the viability, proliferation and migration of HK2 cells receiving different treatments. (**D**–**F**) qRT-PCR analyses of the expression profile of the MALAT1/miR-145/FAK axis in HK2 cells receiving different treatments. GAPDH was used as a control. *P < 0.05 and **P < 0.01. (**G**–**I**) qPCR analysis of MALAT1, miR-145 and FAK expressions in sham group, UUO models and UUO models receiving DHA treatment.

To examine the role and underlying mechanism of DHA in TGF-β1-induced fibrosis, we added DHA to HK2 cells treated with TGF-β1. Western blot analysis revealed that DHA reversed the TGF-β1-induced EMT in HK2 cells (Figure 7A). Then, CCK-8, EdU and migration analyses demonstrated that DHA inhibited the TGF-β1-induced promotion of the viability, proliferation and migration potential of HK2 cells (Figure 7B and 7C). Taken together, these observations demonstrated that DHA alleviated TGF-β1-induced fibrosis.

We next wondered whether the MALAT1/miR-145/FAK axis was involved in the antifibrotic effect of DHA. qPCR analysis demonstrated that DHA inhibited the TGF-β1-induced upregulation of MALAT1 and FAK and restored the TGF-β1-induced suppression of miR-145 expression (Figure 7D–7F). In summary, these results demonstrated that the MALAT1/miR-145/FAK axis was involved in the antifibrotic effect of DHA *in vitro*.

### The MALAT1/miR-145/FAK axis is involved in renal fibrosis in UUO mouse models

To further confirm the involvement of the MALAT1/miR-145/FAK pathway in the antifibrotic effect of DHA, we first generated UUO mouse models. HE staining analysis revealed that DHA exerted antifibrotic effects in the UUO (Supplementary Figure 3). Furthermore, IHC staining analysis showed that DHA inhibited α-SMA and ECM deposition in the UUO (Supplementary Figure 4C and 4F). Taken together, these observations revealed the antifibrotic effect of DHA *in vivo*.

Next, we used qPCR analyses to assess the MALAT1/miR-145/FAK pathway in UUO mouse models treated with DHA. qPCR analyses demonstrated that DHA inhibited the increase in MALAT1 and FAK expression and restored the inhibition of miR-145 expression in both the UUO (Figure 7G–7I). IHC staining analysis further confirmed that DHA inhibited the increase in FAK expression in UUO mouse models (Supplementary Figure 4I). In summary, these results demonstrated that the MALAT1/miR-145/FAK axis was involved in the antifibrotic effect of DHA *in vivo*.

### m^6^A modification participates in the upregulation of MALAT1 in renal fibrosis

**Figure 8:**
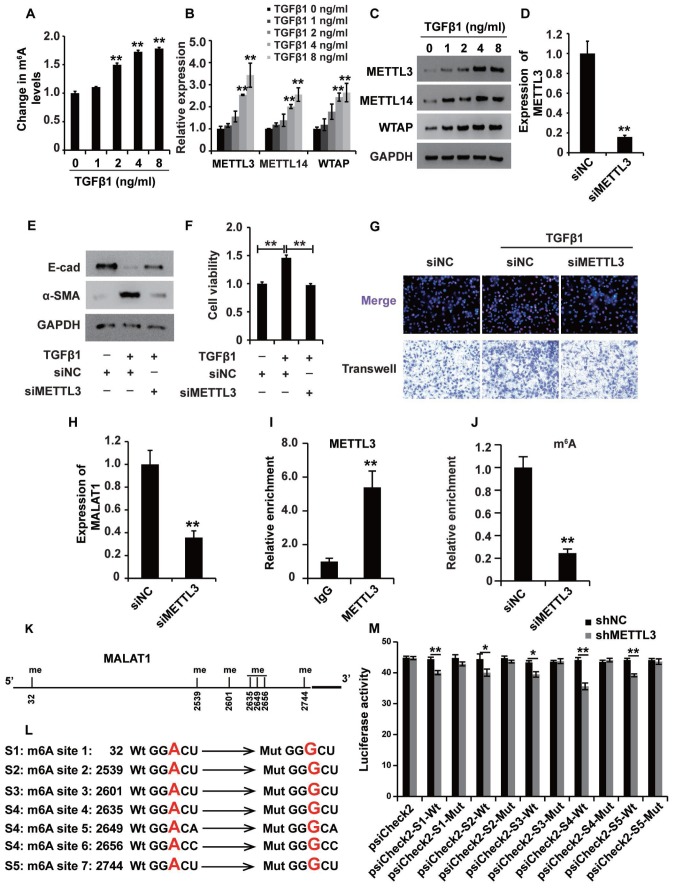
**mA modification participates in the upregulation of MALAT1 in renal fibrosis.** (**A**) ELISA was used to measure the m^6^A levels in HK2 cells treated with TGF-β1 at different concentrations for approximately 48 h. (**B** and **C**) qPCR and western blot analyses of METTL3, METTL14 and WTAP in HK2 cells treated with TGF-β1 at different concentrations for approximately 48 h. (**D**) qPCR analyses of METTL3 expression in HK2 cells transfected with siMETTL3 or siNC for approximately 48 h. (**E**) qPCR analyses of E-cad and α-SMA expressions in HK2 cells treated with siNC, siNC+TGF-β1 and siMETTL3+TGF-β1 for approximately 48 h. (**F** and **G**) CCK8, EdU and cell migration analyses of the viability, proliferation and migration potential of HK2 cells treated with siNC, siNC+TGF-β1 and siMETTL3+TGF-β1 for approximately 48 h. (**H**) qPCR analyses of MALAT1 expression in HK2 cells transfected with siMETTL3 or siNC for approximately 48 h. (**I**) METTL3 RIP-qPCR analysis of MALAT1 in HK2 cells. (**J**) m6A RIP-qPCR analysis of MALAT1 in HK2 cells transfected with siMETTL3 or siNC for approximately 48 h. (**K**) the predicted m^6^A sites (with high confidence) in MALAT1. (**L**) the mutated m^6^A sites of MALAT1. (**M**) the inhibitory role of shMETTL3 was attenuated in the luciferase activity inserted the MALAT1 with mutant type (Mut) m6A sites. GAPDH was used as a control. *P < 0.05 and **P < 0.01.

The m^6^A modification was reported to play important roles in a variety of cellular processes by affecting RNA stability and protein translation efficiency [34]. First, the ELISA was used to detect the m^6^A level in HK2 cells treated with TGF-β1, revealing that TGF-β1 elevated the m^6^A level in HK2 cells (Figure 8A), suggesting that m^6^A modification occurred in HK2 cells treated with TGF-β1. qPCR and western blot analyses showed that the m^6^A methyltransferases METTL3, METTL14 and WTAP were upregulated in HK2 cells treated with TGF-β1 (Figure 8B and 8C).

To dissect the role of m^6^A modification in HK2 cells treated with TGF-β1, we first knocked down the expression of METTL3 (Figure 8D), which is the key m^6^A methyltransferase. Western blot analysis revealed that suppressing METTL3 could attenuate the TGF-β1-induced EMT in HK2 cells (Figure 8E). Then, CCK8, EdU and cell migration assays were performed to confirm that suppressing METTL3 inhibited the viability, proliferation and migration potential of HK2 cells (Figure 8F and 8G). Collectively, these observations revealed that m^6^A modification played a crucial role in TGF-β1-treated HK2 cells.

Next, we wondered whether m^6^A modification was involved in the upregulation of MALAT1 in HK2 cells treated with TGF-β1. qPCR analysis demonstrated that suppressing METTL3 inhibited the expression of MALAT1 in HK2 cells (Figure 8H). METTL3 immunoprecipitation (RIP)-qPCR analysis revealed a 5.3-fold enrichment in the METTL3 antibody levels of MALAT1 in HK2 cells (Figure 8I). In addition, m^6^A RIP-qPCR analysis illustrated that suppressing METTL3 inhibited the enrichment of MALAT1 m^6^A antibody levels in HK2 cells by 4-fold (Figure 8J). To discover the m^6^A sites of MALAT1, we firstly predicted the m6A sites in MALAT1 using http://www.cuilab.cn and introduced the luciferase analysis to detect the m^6^A sites in MALAT1. There were 7 m^6^A sites (with high confidence) in MALAT1 (Figure 8K). Then, we mutated the m^6^A sites in MALAT1(Figure 8L) and the results of luciferase analysis demonstrated that shMETTL3 inhibited the activity of luciferase reporter which inserted the MALAT1 with wild type (Wt) m^6^A sites and meanwhile, the inhibitory role of shMETTL3 was attenuated in the luciferase inserted the MALAT1 with mutant type (Mut) m^6^A sites (Figure 8M). In summary, these results revealed that m^6^A modification participated in the upregulation of MALAT1 in renal fibrosis.

## DISCUSSION

ON is not uncommon in the clinic and is often seen in urology departments. Long-term mechanical urinary obstruction caused by, for example, congenital anatomic abnormalities in children, urinary stones in young adults or malignancies in older adults, leads to CKD. The early detection of urinary obstruction can save renal function and improve patient outcomes. Serum creatinine (Scr) and glomerular filtration rate (GFR) are widely used for evaluating renal function, and imaging examinations provide visual evidence of obstruction. However, these methods are not sufficiently sensitive for the early detection of ON, especially in asymptomatic patients whose severe hydronephrosis and renal dysfunction are detected by accident. Apart from this, the pain caused by urologic obstruction is not proportional to the severity of the disease, which is often neglected. Therefore, more sensitive biomarkers are necessary for the earlier warning and detection of ON.

Renal fibrosis is initiated and sustained by a variety of prosclerotic properties, and TGF-β1 is known as the most potent inducer of fibrosis [45, 46]. Although its biological roles in different cell types and biological scenarios are controversial, TGF-β1 plays important roles in the induction of fibrosis [47, 48]. TGF-β1 induces fibrosis by inducing EMT and promoting the expression of ECM proteins [13]. In our study, TGF-β1 induced EMT and increased ECM protein deposition in HK2 cells. Furthermore, the growth, mobility and dysfunction of tubular cells is the leading cause of CKD [49]. Thus, assessing tubular cell functional properties, such as their viability, proliferation and migration abilities, is important to better understand CKD progression and will ultimately lead to improvements in the monitoring techniques and therapeutic interventions for CKD. In the present study, TGF-β1 promoted the viability, proliferation and migration of HK2 cells by activating the MALAT1/miR-145/FAK pathway.

Recent research has shown that lncRNAs are involved in a variety of cellular processes and can be sensitive biomarkers for diseases. Advances in transcriptome analysis by RNA-seq technology have enabled the elucidation of lncRNA functions in renal fibrosis [50]. Several lncRNAs, such as MEG3, H19 and HOTAIR, were reported to play important roles in mediating TGF-β and renal fibrosis [45–53]. MALAT1 was reported to affect the cellular processes induced by TGF-β, such as EMT, ECM protein deposition, cell growth and cell mobility [43]. In our study, we demonstrated for the first time that TGF-β1 promoted MALAT1 expression and that knocking down MALAT1 expression inhibited TGF-β1-induced EMT, ECM deposition and the viability, proliferation and migration of HK2 cells.

In recent years, an increasing number of reports have revealed that a variety of lncRNAs act via the regulatory mechanism described by the ceRNA hypothesis [44]. The corresponding lncRNAs, miRNAs and mRNAs participate in the regulatory pathway, and any perturbation of this pathway alters the cellular processes and physiological and pathological progression of human diseases [54]. A variety of lncRNAs, including SBF2-AS1, SNHG7, TBILA, UCA1, ATB, and MALAT1, play important roles in TGFβ-treated cells via ceRNA mechanisms (43). Xiang Y and colleagues demonstrated that MALAT1 modulates the TGF-β1-induced EMT of endothelial progenitor cells (EPCs) by regulating TGFBR2 and SMAD3 via miR-145 [55]. In our study, we found that MALAT1 activated FAK expression by sponging miR-145. In addition, we revealed that activating the MALAT1/miR-145/FAK ceRNA network could increase TGF-β1-induced renal fibrosis *in vitro* and significantly altered MALAT1, miR-145 and FAK expression *in vivo*.

Furthermore, we investigated whether m^6^A methylation was involved in the upregulation of MALAT1 in TGF-β1-treated HK2 cells. First, the m^6^A level was elevated, and we confirmed that METTL3 was upregulated in TGF-β1-treated HK2 cells. METTL3 was shown to function and positively regulate MALAT1 in TGF-β1-treated HK2 cells. Thus, we revealed that m^6^A modifications can modulate MALAT1 in TGF-β1-treated HK2 cells and possibly affect the MALAT1/miR-145/FAK pathway in renal fibrosis.

Currently, surgical treatment is the top option for relieving urological obstruction. Nevertheless, the timing of surgical treatment and the effect of early surgical treatment remain controversial in the following three groups of patients: (1) patients who are not in good physical condition and cannot tolerate surgery; (2) patients whose obstruction cannot be thoroughly removed and are prone to relapse; and (3) patients with congenital ON. Therefore, antifibrotic drugs are necessary for these patients. Fortunately, DHA has been shown to effectively attenuate renal fibrosis caused by ON through the MALAT1/miR-145/FAK axis *in vivo* and *in vitro*. Unfortunately, DHA has not been used clinically to treat patients with renal fibrosis caused by ON.

In conclusion, the present study reveals that the expression profile of MALAT1/miR-145/FAK may be a potential biomarker for the diagnosis and treatment of ON-induced renal fibrosis in CKD. In addition, the m^6^A modification is involved in the regulation of MALAT1. Moreover, DHA can attenuate ON-induced renal fibrosis through the MALAT1/miR-145/FAK axis.

## MATERIALS AND METHODS

### Cell culture and reagents

Human renal proximal tubular epithelial cells (HK2 cells, human kidney-2: ATCC CRL-2190) were purchased from American Type Culture Collection (ATCC, Manassas, VA, USA) and cultured in Dulbecco’s Modified Eagle’s Medium with F12 (Invitrogen, Carlsbad, USA) supplemented with 10% heat-inactivated foetal bovine serum, 100 U/ml penicillin and 100 U/ml streptomycin at 37°C in a humidified atmosphere containing 5% CO_2_.

TGF-β1 was purchased from PeproTech (PeproTech, Rocky Hill, USA) and dissolved in dimethyl sulfoxide (DMSO). DHA (>98% purity; MW 284.35) was purchased from Shanghai Macklin Biochemical Co., Ltd. (Shanghai, China) and dissolved in DMSO.

### Human specimens and animal models

This study was approved and supervised by the Research Ethics Committee of Xiangya Hospital, Central South University (no. 201703525 and 201703526).

Clinical specimens were collected from patients undergoing surgical resection at Xiangya Hospital, Central South University; the samples were then quickly frozen in liquid nitrogen and stored at -80°C for no more than 1 year. Informed consent agreements were signed by all patients.

For the unilateral ureteral obstruction (UUO) model, male C57BL/6J mice at 8 weeks of age (20–22 g body weight) were first anaesthetized with pentobarbital sodium (50 mg/kg) via intraperitoneal injection. Then, the left ureter was ligated using 3-0 silk and a left lateral incision. Sham-operated C57BL/6J mice were used as the experimental controls.

### RNA interference (RNAi)

Stealth RNAi oligonucleotides (siRNAs) specifically targeting MALAT1 and FAK were designed and synthesized by GenePharma (Shanghai, China). The siRNA sequences were as follows: siMALAT1-1: 5′-GGACAACAGUACACGCAUATT-3′; siMALAT1-2: 5′-GCCACCUACAUUAAAGCUATT-3′; siMALAT1 -3: 5′-GACCAGACCCTACCGGTCATTTATUATT-3′; siFAK-1: 5′-GCUAUGGUGAACACCCUAATT-3′; siFAK-2: 5′-GAACUGGGAUUCACAUCGA-3′; siFAK-3: 5′-GGAUAACGAUGGCUACUCA-3′; and negative control siRNA: 5′-GACCTACAACTACCTATCA-3′.

### Transient transfection

HK2 cells were plated in 12-well plates at a density of 1.0×10^5^ cells per well. The next day, upon reaching approximately 80~90% confluence, the cells were transfected with Lipofectamine 2000 (Invitrogen) according to the manufacturer’s instructions. After 48 h, the HK2 cells were used for further research.

### Total RNA isolation and qRT-PCR

Total RNA was isolated from cultured cells and tissues using TRIzol (Invitrogen, Carlsbad, CA, USA) according to the manufacturer’s instructions. A NanoDrop ND-1000 spectrophotometer (Thermo Scientific) was employed to determine the total RNA concentrations. Reverse transcription was performed with 1 μg of total RNA and a PrimerScript RT reagent kit with gDNA Eraser (TaKaRa, Dalian, China). qPCR was performed with a SYBR Green I array (Donghuan Biotech, Shanghai, China) and the Prism 7500 SDS system (Applied Biosystems, Thermo Fisher Scientific). Each sample was measured in triplicate. The data were analysed using the 2^-∆∆Ct^ method. Relative mRNA and microRNA expression levels were normalized to those of GAPDH or U6 small nuclear RNA, and arbitrary units were used to show the normalized relative gene expression levels. The primers were designed and synthesized by Sangon Biotech (Shanghai, China). The qRT-PCR primers for MALAT1 were as follows: forward: 5′-TTACCTGGGGAACCCCGACC-3′ and reverse: 5′-TGGTGAAGGATGAGGGCTCGT-3′. The qRT-PCR primers for FAK were as follows: forward: 5′-TTACCTGGGGAACCCCGACC-3′ and reverse: 5′-TGG TGAAGGATGAGGGCTCGT-3′. The primers for E-cadherin were as follows: forward: 5′-GCTTCAGCAAAGACAACGAG-3′ and reverse: 5′-GTGTAATGCAGGACCACAGC-3′. The primers for ZO1 were as follows: forward: 5′-CGGGTCTACGCCTACGTCTTTGAACACCGTGCTTC-3′ and reverse: 5′-CACAGGTCTGAGCAGCGATCCTGCTTGCTG-3′. The primers for vimentin were as follows: forward: 5′-CGGGTCTACGCCTACGTCTTTGAACACCGTGCTTC-3′ and reverse: 5′-ATGGGTGAAGCCTGGGCAGGTG-3′. The primers for α-SMA were as follows: forward: 5′-CCTGTCCACACGGGTGAACT-3′ and reverse: 5′-CACCAGGCCTAGCATTCATTG-3′. The primers for β-actin were as follows: forward: 5′-ACTACCTGAGCACCCAGTCC-3′ and reverse: 5′-CACAGGTCTGAGCAGCGATCCTGCTTGCTG-3′. β-Actin was used as a control.

### Western blot analysis

HK2 cells receiving different treatments were harvested and lysed with RIPA lysis buffer (Beyotime). Protein concentrations were measured using a BCA Protein Assay Kit (Donghuan Biotech, Shanghai, China). Equal amounts of protein lysates were separated by electrophoresis on a 10% SDS-polyacrylamide gel and transferred onto polyvinylidene difluoride (PVDF) membranes (Millipore). After blocking in 5% fat-free milk dissolved in PBST, the blots were incubated with different primary antibodies at 4°C overnight. The next day, the primary antibodies were removed, and the blots were incubated with an anti-IgG horseradish peroxidase-conjugated secondary antibody. GAPDH was measured as a control. The antibodies used in our study were E-cadherin (Abcam, UK; dilution of 1:2000), vimentin (Abcam, UK; dilution of 1:2000), N-cadherin (Abcam, UK; dilution of 1:2000), FAK (Cell Signaling Technology, USA; dilution of 1:2000), GAPDH (Abcam, UK; dilution of 1:5000) and a secondary fluorescent goat anti-rabbit antibody (Abcam, UK; dilution of 1:4000). Immunoreactivity was visualized using an ECL western blot system (Donghuan Biotech, Shanghai, China), and the data were analysed using Gel-Pro Analyzer Software.

### CCK-8 assay

The viability of HK2 cells receiving different treatments was measured using Cell Counting Kit-8 (Donghuan Biotech, Shanghai, China). HK2 cells were plated in 96-well plates at a density of 4000 cells/well in triplicate. After the cells received the different treatments, 10 μL of CCK-8 reagent was added to each well. After incubation at 37°C for 4 h, the absorbance at 490 nm was measured using a microplate reader. Then, the cell growth curves were generated, and arbitrary units were applied to show the normalized relative differences among the different groups.

### Ethynyl-2ʹ-deoxyuridine (EdU) assay

The proliferation of HK2 cells receiving different treatments was detected using an EdU assay kit (Donghuan Biotech, Shanghai, China). Briefly, HK2 cells receiving different treatments were plated in 48-well plates at a density of 1.0×10^5^ cells per well. The next day, EdU was added to the cells and incubated for 2 h at 37°C. Then, the cells were fixed with 4% formaldehyde for 25 min at room temperature (RT), permeabilized with 0.5% Triton X-100 for 25 min at RT and incubated with the staining solution for 30 min at RT. Hoechst 33342 was used to indicate the cells by staining their DNA. Immunofluorescence was detected under a fluorescence microscope (ZKX53, Japan).

### Cell migration assay

The migration potential of HK2 cells receiving different treatments was measured using Transwell chambers (Corning). Briefly, HK2 cells receiving different treatments were plated in 24-well plates at a density of 2.5×10^5^ cells per well with medium containing 1% FBS; medium containing 10% FBS was added to the lower wells. The next day, the cells on the upper side of the membrane were removed. Then, the cells on the lower side were washed with PBS, fixed with 95% alcohol, stained with a 0.1% crystal violet solution, and air dried. The results of five random fields were imaged using a fluorescence microscope (Olympus, Japan).

### m^6^A analysis and RNA immunoprecipitation (RIP)

Total RNAs were extracted with Trizol (Donghuan Biotech, Shanghai, China) according to the protocol. The levels of total m^6^A were detected in equal amount (300 ng) of total RNAs with the m^6^A RNA methylation detection kit (Epigentek, Farmingdale, NY).

RNA immunoprecipitation (RIP) analysis was performed with an RNA-Binding Protein Immunoprecipitation Kit (Millipore) according to the protocol. Briefly, 5 μg of anti-m6A antibody, 500 μg of cellular RNA, and 20 μl (in 50% slurry) protein-A/G sepharose were incubated in RIP buffer [150 nM NaCl, 0.1% NP-40, 10 mM Tris. HCl (pH. 7.4)] plus 1 U/μl RNasin in 200 μl at 4°C overnight. The beads were washed with the RIP buffer for 5 times. RNA isolated from the IP beads were subjected to reverse transcription (RT) followed by real-time, quantitative (q)PCR analysis.

### Luciferase reporter assay

Full-length MALAT1 and FAK-3′UTR sequences were amplified by PCR and cloned into the psiCHECK-2 plasmid (Promega). The sequence of the putative binding site (miR-145) was replaced with MALAT1-Mutant (MALAT1-Mut) and FAK-3′UTR-Mutant (FAK-Mut). In brief, 293 cells were seeded into 6-well plates at a density of 4 × 10^5^ cells per well. The next day, UCA1 and ZEB1Wt or Mut plasmids and miR-455 mimics were transfected into the cells using Lipofectamine 2000 reagent (Invitrogen) according to the manufacturer’s instructions. After 48 h, the firefly and Renilla luciferase activities were measured using a Dual-Luciferase Assay System (Promega).

### Statistical analysis

The data were analysed using the SPSS software package (version 13.0.0) and expressed as the mean ± standard deviation (S.D.). All experiments were repeated a minimum of three times. Student’s t-test was used to assess the differences between two groups, and one-way analysis of variance was employed to detect the differences between more than two groups. The symbol * represents a statistically significant difference (*P* < 0.05), while ** indicates a highly significant difference (*P* < 0.01).

## Supplementary Material

Supplementary Figures
