# Supplementary material for: m6A-induced lncRNA MALAT1 aggravates renal fibrogenesis in obstructive nephropathy through the miR-145/FAK pathway
Source: Aging (Albany NY). 2020 Mar 23;12(6):5280–99. doi: 10.18632/aging.102950 (PMC7138587; doi:10.18632/aging.102950)
Supplement: Supplementary Figures [file aging-12-102950-s001..pdf]

SUPPLEMENTARY FIGURES

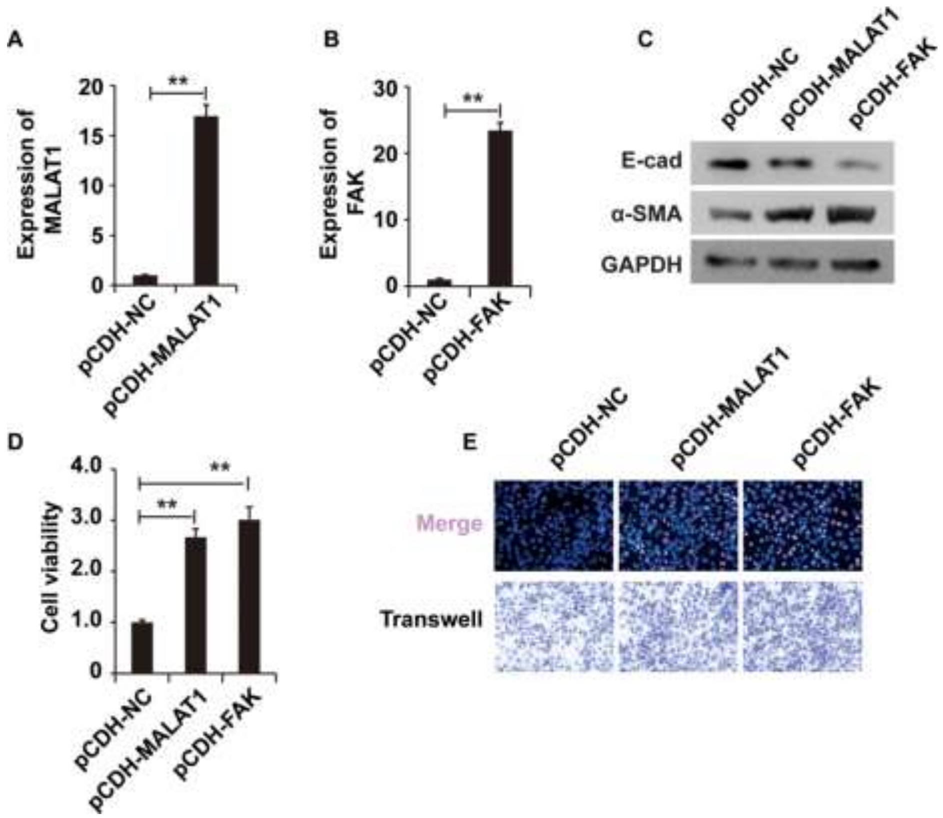

Supplementary Figure 1. Over-expressed MALAT1 and FAK in HK2 cells and the qPCR, CCK8, EdU and Transwell outcomes. (**A**) qRT-PCR analysis of MALAT1 expression in HK2 cells transfected with pCDH-MALAT1 or pCDH-NC. (**B**) qRT-PCR analysis of FAK expression in HK2 cells transfected with pCDH-FAK or pCDH-NC. (**C**) Western blot analyses of E-cad, α-SMA and GAPDH expression in HK2 cells treated with overexpression of MALAT1 or FAK. (**D** and **E**) CCK-8 and EdU of the viability and proliferation of HK2 cells treated with overexpression of MALAT1 or FAK.

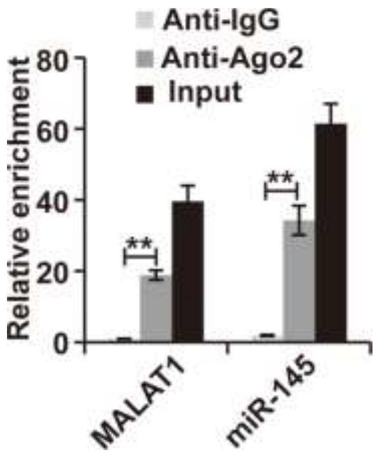

Supplementary Figure 2. Results of RIP showing that MALAT1 and miR-145 were more abundant in the Ago2 pellet than in the control IgG pellet.

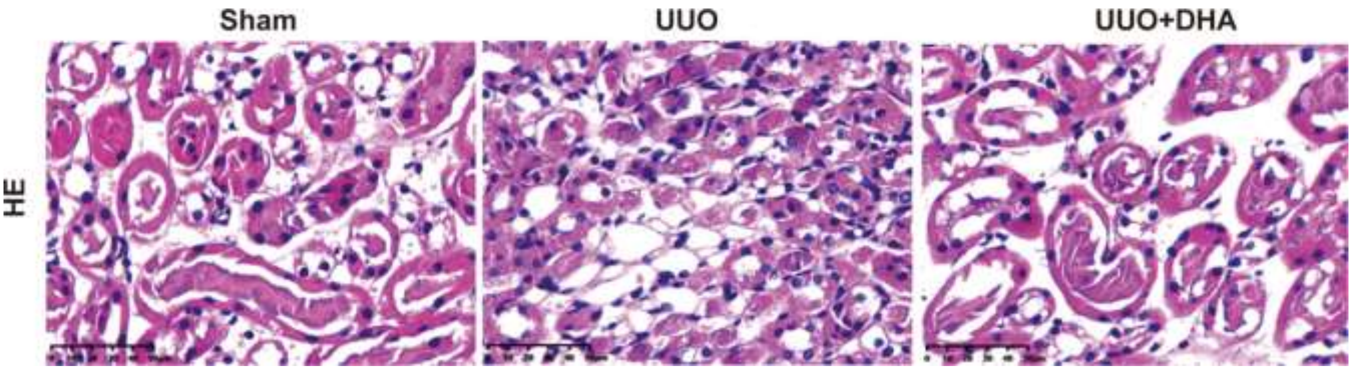

Supplementary Figure 3. HE staining of sham group, UUO models and UUO models receiving DHA treatment.

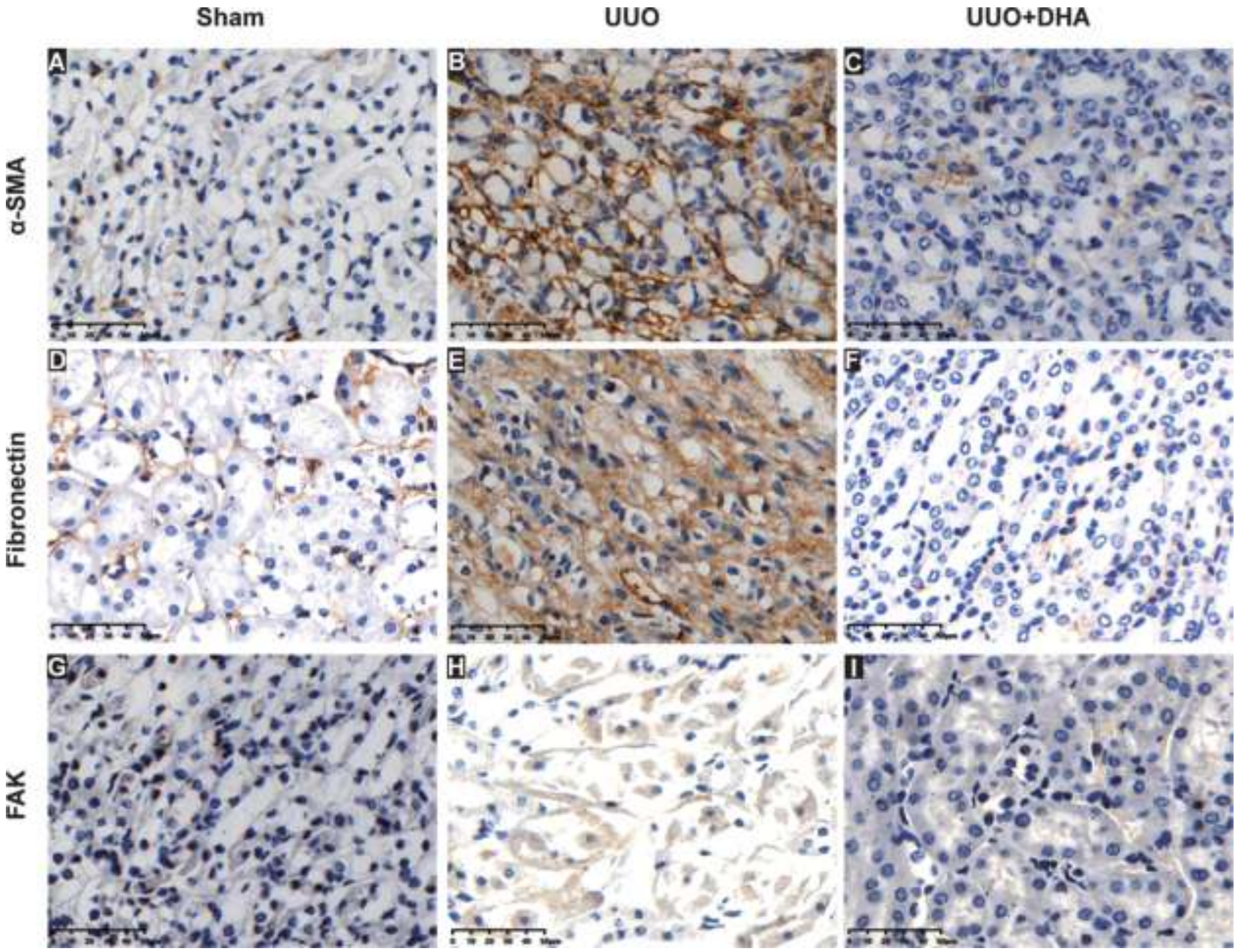

Supplementary Figure 4. IHC staining analysis of mouse models with DHA treatment. (**A**–**C**) IHC of α-SMA in Sham group, UUO group and UUO + DHA group. (**D**–**F**) IHC of Fibronectin in Sham group, UUO group and UUO + DHA group. (**G**–**I**) IHC of FAK in Sham group, UUO group and UUO + DHA group.
